# The Carrel-Dakin and Allied Methods

**Published:** 1919-06-07

**Authors:** 


### THE WAR AND SURGICAL PROGRESS.

### II.—The Carrel-Dakin and Allied Methods.

ALTHOUGH known to have produced many brilliant results and supported by surgeons of world-wide fame, the Carrel-Dakin treatment has been opposed by experts just as eminent. In a previous article we attempted to explain its essentially secondary position with regard to wounds of slight nature or received in the earliest stages. With such lesions thorough cleansing, followed by immediate suture, is held in high favour, and is undoubtedly the ideal method, but becomes impracticable if septic processes once gain the upper hand. Nevertheless, a number of experienced authorities now condemn the use of all antiseptics on the ground that they still further injure damaged tissue, and in reality give little or no direct aid in either the process of repair or the prevention of infection.

This disagreement among so many able men suggests immediately that each one has concentrated upon a particular aspect of the problem, each one has unravelled one section only without perceiving the whole situation in its true perspective. Much depends on the character of the injury, on the period at which it comes under observation, but more depends upon the judgment and skill with which both the general and minute requirements of each case are realised, and methods exactly appropriate are applied. In any case, it may be said that the decision for or against the Carrel-Dakin treatment can scarcely be reached by any individual surgeon without personal experience of its employment. Its known degree of success is such as to warrant a trial under all circumstances deemed from past records likely to be favourable.

### THE ESSENTIAL TECHNIQUE.

Starting with those cases where primary or delayed primary suture has been found impossible for any of the reasons previously described, one can briefly outline the chief procedures. Originally under this method all wounds, however early, were treated as suspects, and clinical appearances, however favourable, were to be corroborated by bacteriological control before any attempt at closure was made. The present view is mainly that in early cases wounds are to be given the opportunity to prove their infected nature before antiseptic treatment is begun. Should sepsis become obviously present, then use must be made of the instillation or allied methods.

The affected area is freely exposed by incision, bringing all recesses into view and tending to convert any irregular laceration into an open chamber wound, at the same time preserving as far as possible all tendonous, nervous, or vascular structures. Radical removal of all foreign bodies, contaminations, the immediate tracks of projectiles, etc., is, of course, essential to preliminary wound purification. Control of hæmorrhage by plain catgut ligature and of oozing by heat must be carefully accomplished, since, apart from interference with sterilisation, blood considerably complicates the making of bacteriological controls. *X*-rays are invaluable in the theatre both for locating metallic fragments and detecting fractures or loose bone. A neutral oleate of soda is advised for preliminary cleansing, or for removing contaminations from the surrounding skin. The wound area thus prepared is ready for introduction of hypochlorite solution.

Actual instillation and dressing procedures are by now too well known to require detailed description, and it is only necessary to recall a few of the major points. The solution must be renewed every two hours at least, since by that time its efficiency is greatly decreased; but no removal of dressings is necessary. It may be coloured faintly pink with potassium permanganate, not only to distinguish it from other irrigating fluids, but in some measure to protect it from decomposition. Direct smears of exudate from various parts of the wound are made on alternate days, and the time for closure governed by laboratory reports. Films are made by the methods customary with simple blood examination after instillation has been discontinued for two hours. They must be of as nearly as possible constant density, and preferably taken by the surgeon himself, to provide a constant personal equation in technique. Time for suture has not arrived until the proportion of bacteria is as low as one organism to five or six fields of closely set leucocytes under the $\frac{1}{12}$-inch objective of the microscope. If closure be even then followed by rise of temperature or evidence of local disturbance, reopening must be made without delay and instillation repeated or foreign matter, if present, removed. In some cases bacteria may be imprisoned in scar or partially healed tissue, and, as recently indicated by Sir Kenneth Goadby, be set free when operation and final manipulation of the wound edges are attempted. This undoubtedly accounts for many of the unfavourable results encountered.

### METHODS OF CLOSURE.

There are several ways by which the final step may be taken. If the skin edges be freely movable and cicatrisation has not begun excellent results may be obtained by mere strapping with adhesive plaster, or, with widely gaping wounds, more powerful elastic tension will greatly reduce the amount of space left to cicatrise if it does not actually bring the surfaces immediately into contact. If suture be the method selected, a general anæsthetic should be given, and the whole wound and skin area freshened with the knife, muscles, nerves and special tissue sutured separately and, as bacteriological examination has or should have shown requisite freedom from organisms, the closure may be completed without artificial drainage. It has been found possible during this stage to introduce adipose, muscular, or even bone grafts, when extensive removal of tissues has previously occurred. In the official American report on their own experiences it is stated that, "Wounds associ-

**War and Surgical Progress.**—(*continued*).

ated with fractures may be closed, as a rule, the same as wounds of the soft parts. Even in badly crushed limbs, if asepsis can be secured in a few days closure may be affected without any anxiety regarding the bony element of the problem. It will take care of itself," and the surgeons of that country have given the method a more than searching trial.

### Claims to General Application.

Carrel himself claims for his invention merely a great saving in life and limb, particularly with the traumatic injuries of war, in conditions which previously would have been considered absolutely impossible. "Domination in the frequency and intensity of general complications; diminution in the number of amputations; diminution in the length and cost of treatment," are perhaps chief among the points he makes. With regard to the first, records of the hospital of Compiègne show an extraordinarily low mortality among cases who, after their journey from the advanced dressing stations, survived long enough, namely twenty-four hours, for the process of sterilisation to take effect. For the second one may take the words of Surg. Lt.-Com. W. S. Bainbridge, U.S.N., who has made in the *Medical Bulletin* an exhaustive and excellent survey of the French results, and from whose notes we quote freely.

Suppression of infection prevented the complication of lymphangitis, abscess and purulent tracts, diminishing the number of amputations. In one year (at Compiègne) only three abscesses were observed. Where the extent and character of the lesions did not allow of speedy sterilisation they did allow of a great deal of local improvement. This favoured the conservative management of many wounds ordinarily calling for amputation. From December 1915 to October 1916 only 23 amputations were performed, and where resection of joints was the usual course the simpler expedient of arthrotomy and sterilisation sufficed to save the limb. The amputations were due mostly to such extensive destruction of tissue that nothing else was possible. In only three cases was the cause sepsis.

Also it is obvious that reduction in the septic process must greatly accelerate the possibility of suturing nerves, tendons, and specialised tissues, thus making for great improvement in the ultimate functional utility of the injured parts. One could give an almost endless list of surgeons who have given favourable reports on this technique as used in our own army, and, to take one example, Crisp-English and Kelly, referring to experiences with the Salonika forces give the opinion that, although many other forms of treatment have been in use there, such as hypertonic saline, salt packs, etc., none has shown the same consistency of results, the rapid sterilisation of the wound, and its early secondary suture such as occur with Carrel's method.

### Possible Objections.

Objections have been raised chiefly against the particular antiseptic solution rather than against the technique itself. Three points of weakness are suggested, (*a*) the irritation of hypochlorite solutions to the skin, (*b*) their brief efficiency, (*c*) the necessity for uninterrupted contact of the fluid with every recess of the wound. Dakin suggested the use of dichloramin-T in oil to overcome such difficulties, but even then value is lost because in the instance of (*c*), the oily fluid does not appear to reach the remoter parts, and also does not efficiently dissolve out necrotic tissue from deep wounds. In fact, although Carrel and Hartmann themselves published in 1918 accounts of a successful modification in which a chloramin paste containing 8% of sodium stearate and 4 to 15 parts per thousand of chloramin-T was used in superficial or slight septic lesions, yet consensus of opinion is tending strongly toward the retention of the hypochlorite solution and the original instillation methods. The antiseptic itself is very low in cost of production and apparatus readily adapted to both local and institutional use, and even if the Carrel-Dakin is still not the ideal procedure, its employment has revealed invaluable truths. The paramount necessity of asepsis, reinforced or ultimately attained by means of such antiseptic methods, is proving to be one of the great lessons of these times. The exact type of antiseptic and the precise mode of its employment may be still open to improvement, but at the moment such matters are largely controlled by individual preference and experience. The principle holds good, and for routine administration one finds it hard for the moment greatly to improve upon the essential idea of the Carrel-Dakin method.

